# That Mosquito Fallacy

**Published:** 1901-12

**Authors:** 


### THAT MOSQUITO FALLACY.

Like the thief in the community to whose charge every bad thing is laid, the mosquito has now to be responsible not only for malaria, but for yellow fever! Of all the wild and senseless things I have ever heard seriously proposed, not even excepting homœopathy and "Christian science," the assertion that the mosquito is the sole means of spreading yellow fever is the wildest and most dangerous. The assertion is made in the face of the accumulated and overwhelming evidence to the contrary, evidence abundant and forthcoming, offered by the history of every epidemic which has ever occurred in America of which we have any record. That a mosquito or a fly might, under certain circumstances, communicate yellow fever to a person is possible, but not probable. The advocates, Doty *et al.*, seem to have jumped to a conclusion, based upon a single, or, at most, insufficient observation, and hasten to stullify themselves by contradicting the experience and observation of all the yellow fever physicians of the time when epidemics swept this country with such fatal results. That fellow fever is carried from one point to another, often remote, by infected persons and things; that it may be and is spread by infected air confined in baggage, bales or boxes of merchandise (*fomites*) is a fact as well attested and assured as any fact in the history of medicine. It is upon this knowledge of the facts that all quarantine systems for its exclusion is based, and the recent history of Texas in relation to yellow fever, as well as that of other States, testifies to the soundness of the system.

There are two, certain, ways in which yellow fever is spread: by persons from centers of infection, in whose system the disease is incubating; and by goods or merchandise or any textile, or other matter capable of carrying confined air. The first case at Jackson, Mississippi, in 1878, was caused by a demijohn of rum or brandy, packed in sawdust and shavings in a nailed-up box, which was smuggled in from Cuba *via* some obscure point on the Florida coast. The writer diagnosed the case, and the means whereby it had been introduced was discovered later. An epidemic followed. The disease was introduced into Lake, Mississippi, the same year by a family named Sneed, who refugeed from Vicksburg, both by infected baggage and in the person of Mr. Sneed. Sneed recovered, after lighting up the most fearful and memorable epidemic the South ever suffered. His family all perished. The writer was there. The history of the camp near Memphis the same year—Camp Plunket, I think, it was called—negatives the idea that the mosquito is the means of spreading the disease. I assume that my readers are familiar with the facts I allude to, hence I will not repeat it.

That yellow fever is not *contagious,* and cannot be "caught" as is measles or scarlet fever, for instance, has been often demonstrated under my observation, and it is generally so held. That unsanitary conditions are necessary to the lighting up of an epidemic, as well as a high atmospheric temperature, and that a cleaning up will sometimes prevent a spread even after the infection has been introduced, has also been demonstrated; the last proposition abundantly so, notably, Butler, in New Orleans, in 1862-3.

It would look as if it were useless to cite evidences in support

### BANKRUPT VITALITY

—be it manifested as nervous exhaustion, malnutrition, or anæmia—is the condition that

**GRAY'S** Glycerine **TONIC** Comp.

speedily changes to vigor of nerves, blood and tissues.

THE PURDUE FREDERICK CO.,

No. 15 Murray Street. New York.

of a position so secure as that on which all rational quarantine is based, were it not that as a corollary of the absurd statement that the mosquito is the sole cause of yellow fever epidemics, it is seriously proposed to abolish quarantine, and envelop the Southern States in mosquito netting; to discontinue fumigation of baggage, etc., and detention of persons from an infected place. Hence, the danger I referred to above. It will be a sad day for the Southern States, if mislead by these misguided zealots who have gone off half-cocked—they ever try to experiment. Is there a "nigger" in the woodpile? a bug, a commercial bug, under the chip? We will have something to say on the subject later, when Dr. Oliver's excellent paper reaches us. See proceedings Brazos Valley Medical Association, this issue.

